# MUSCLE: multi-view and multi-scale attentional feature fusion for microRNA–disease associations prediction

**DOI:** 10.1093/bib/bbae167

**Published:** 2024-04-11

**Authors:** Boya Ji, Haitao Zou, Liwen Xu, Xiaolan Xie, Shaoliang Peng

**Affiliations:** College of Computer Science and Electronic Engineering, Hunan University, Changsha 410082, China; College of Computer Science and Electronic Engineering, Hunan University, Changsha 410082, China; College of Information Science and Engineering, Guilin University of Technology, Guilin 541006, China; College of Computer Science and Electronic Engineering, Hunan University, Changsha 410082, China; College of Information Science and Engineering, Guilin University of Technology, Guilin 541006, China; College of Computer Science and Electronic Engineering, Hunan University, Changsha 410082, China

**Keywords:** multi-view learning, graph attention network, deep feature fusion, miRNA–disease associations, multi-scale attention mechanism

## Abstract

MicroRNAs (miRNAs) synergize with various biomolecules in human cells resulting in diverse functions in regulating a wide range of biological processes. Predicting potential disease-associated miRNAs as valuable biomarkers contributes to the treatment of human diseases. However, few previous methods take a holistic perspective and only concentrate on isolated miRNA and disease objects, thereby ignoring that human cells are responsible for multiple relationships. In this work, we first constructed a multi-view graph based on the relationships between miRNAs and various biomolecules, and then utilized graph attention neural network to learn the graph topology features of miRNAs and diseases for each view. Next, we added an attention mechanism again, and developed a multi-scale feature fusion module, aiming to determine the optimal fusion results for the multi-view topology features of miRNAs and diseases. In addition, the prior attribute knowledge of miRNAs and diseases was simultaneously added to achieve better prediction results and solve the cold start problem. Finally, the learned miRNA and disease representations were then concatenated and fed into a multi-layer perceptron for end-to-end training and predicting potential miRNA–disease associations. To assess the efficacy of our model (called MUSCLE), we performed 5- and 10-fold cross-validation (CV), which got average the Area under ROC curves of 0.966${\pm }$0.0102 and 0.973${\pm }$0.0135, respectively, outperforming most current state-of-the-art models. We then examined the impact of crucial parameters on prediction performance and performed ablation experiments on the feature combination and model architecture. Furthermore, the case studies about colon cancer, lung cancer and breast cancer also fully demonstrate the good inductive capability of MUSCLE. Our data and code are free available at a public GitHub repository: https://github.com/zht-code/MUSCLE.git.

## INTRODUCTION

A group of non-coding RNAs, miRNAs or microRNAs are about 22 nucleotides long and control gene transcription by targeting messenger RNA for translational inhibition or degradation [1]. In recent years, numerous studies have elucidated the strong correlation between miRNAs and the occurrence of diverse human diseases [2]. It has been shown by clinical studies that miR-145 and miR-218 are associated with the prognosis of patients with laryngeal cancer [3]. Additionally, the aberrant expression of miR-107 can result in abnormal activity of BACE1 (beta-secretase 1) and contribute to the pathogenesis of Alzheimer’s disease [4]. Studies have demonstrated that miRNAs play vital roles in various biological processes, including cell apoptosis, differentiation and development. The growing attention towards their relationships with complex diseases further underscores their importance [5]. Traditional experimental methods often require extensive laboratory capabilities and analyses, which are accurate, but usually inefficient. Moreover, they are time-consuming and costly, as they involve multiple steps of sample preparation, processing and measurement. Therefore, there is a need for alternative computational methods that can provide fast, accurate and scalable results for predicting the miRNA–disease associations (MDAs) [6, 7].

The prediction of potential MDAs has gained momentum due to the exponential advancement in artificial intelligence. The assumption that miRNAs with similar functions have higher chances of being associated with diseases of similar phenotypes is the basis for developing most of these approaches. For instance, Ning *et al.* [8] presented a computational approach, called AMHMDA, for predicting MDAs. They utilized a fusion attention mechanism and hypernodes to enhance information extraction and prediction accuracy. Experimental results on the HMDD v3.2 demonstrated AMHMDA’s superior performance compared to other methods, validating its robust predictive capability. Li *et al.* [9] introduced HGANMDA, which utilized node- and semantic-layer attentions. They also adopted the similarity assumption and used a bilinear decoder to reconstruct the miRNA–disease connections.

In addition, computational approaches based on matrix decomposition techniques that commonly used to predict MDAs were also developing rapidly. For instance, Chen *et al.* [10] introduced the neighborhood constraint matrix completion method called NCMCMDA, for MDAs prediction. NCMCMDA incorporated neighborhood constraint and matrix completion technology and effectively utilized similarity information to aid prediction, demonstrating superior performance compared to previous computational methods. Chen *et al.* [11] introduced MDHGI, a computational model of matrix decomposition. Integrating various similarity measures and a sparse learning method, MDHGI effectively utilized matrix decomposition before constructing the heterogeneous network, significantly enhancing prediction accuracy. Ha *et al.* [12] introduced a computational framework called SMAP, which also utilized a matrix factorization model and integrated comprehensive similarity measurements for identifying MDAs. SMAP incorporated similarity constraints and demonstrated strong AUCs, underscoring the effectiveness of the matrix factorization approach.

**Figure 1:**
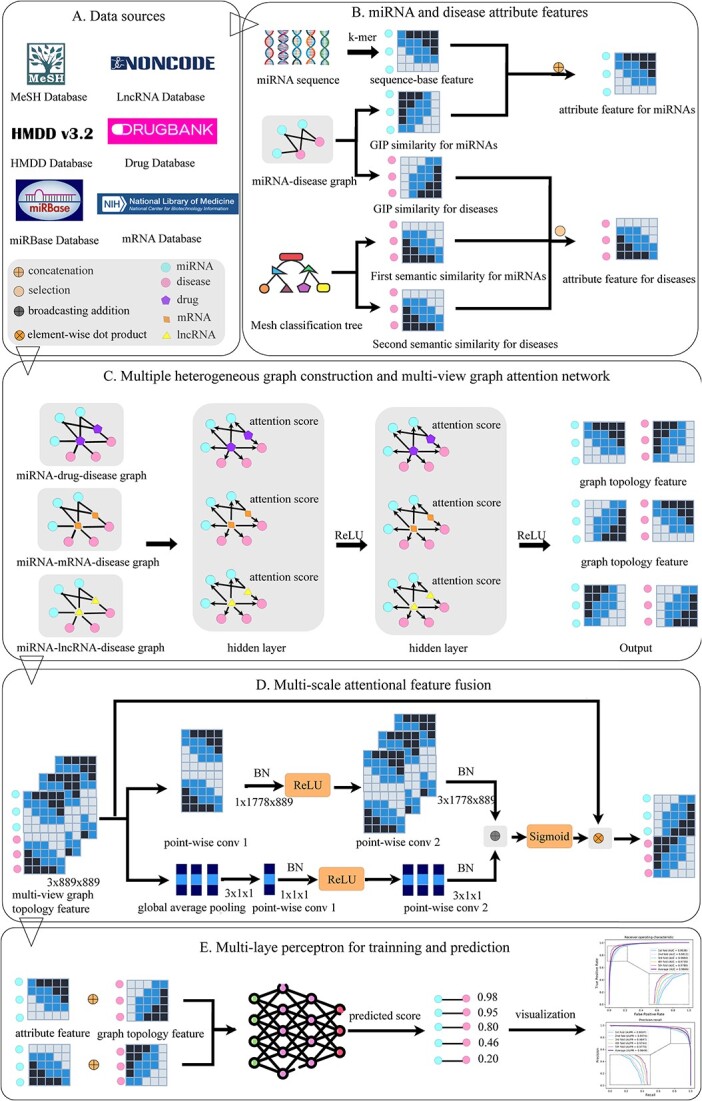
The flowchart of MUSCLE. (**A**) Data sources and some symbols in this study. (**B**) The computation and integration for the prior attribute features. (**C**) Multiple heterogeneous graph construction and multi-view graph attention network for graph topology feature extraction. (**D**) Multi-scale attentional feature fusion mechanism for efficiently fuse these multiple graph topology features. (**E**) MLP for training and prediction with attribute and graph topology features.

To date, despite the variety of computational methods that have been used to predict MDAs, it is important to note that many of them do not take a holistic perspective and only concentrate on isolated miRNA and disease objects, thereby ignoring that human cells are responsible for multiple relationships. Moreover, the efficient fusion method of multi-source features and the incorporation of prior domain knowledge are very important advantages to the model’s prediction ability and application scenarios. On this basis, we proposed MUSCLE for predicting potential associations between miRNAs and diseases. The architecture diagram corresponding to MUSCLE was displayed in Figure 1. Specifically, we first combined multi-source information to construct a multi-view graph, including miRNA–drug–disease graph, miRNA-messenger RNA (mRNA)-disease graph and miRNA-long non-coding RNA (lncRNA)–disease graph. Then, the graph attention neural network was utilized to learn the graph topology features for each view. Next, a multi-scale feature fusion module was designed for efficiently fusing these topology features. In addition, the prior attribute knowledge of miRNAs and diseases was simultaneously added to achieve better prediction results and solve the cold start problem. Finally, the learned representations were concatenated and put into a multi-layer perceptron (MLP) for end-to-end training and predicting. Specifically, the main contributions of MUSCLE are summarized: (i) we utilize the relationship between miRNAs and various biomolecules to built a multi-view graph, and utilize graph attention network to capture graph topology features for each view. (ii) Based on the motivation that a more efficient feature fusion strategy can improve the predictive ability of the model, we design a multi-scale feature fusion module for efficiently fusing multiple topology features by incorporating the local context into the global context within the attention module. (iii) The prior attribute knowledge of miRNAs and diseases was further added to achieve better prediction ability and solve the cold start problem. (iv) Our method shows excellent predictive performance. Each module is tuned to the optimal, and the case studies fully prove the powerful inductive ability of MUSCLE.

## MATERIALS AND METHODS

### Data sources

We obtained MDAs from the Human MicroRNA Disease Database (HMDD v3.2) [13], the latest comprehensive repository for human MDAs, encompassing a broader spectrum of experimentally supported associations. Finally, 12,446 experimentally confirmed MDAs including 901 miRNAs and 877 diseases were selected in this study. We also obtained 269 drug-miRNA pairs and 17,414 drug-disease pairs from the DrugBank database [14] to construct the heterogeneous miRNA–drug–disease graph. From the US National Library of Medicine database, we also obtained 5186 pairs of miRNA-mRNA pairs and 8958 mRNA–disease pairs to construct the heterogeneous miRNA–mRNA–disease associations. Furthermore, we also obtained 8634 lncRNA-miRNA pairs and 874 lncRNA–disease pairs to construct the heterogeneous miRNA–lncRNA–disease graph from the NONCODEV5 database [15]. Finally, an equal number of randomly selected non-MDAs were used as a negative control.

### Sequence-based attribute feature for miRNAs

To capture a comprehensive representation of miRNA features, we incorporated miRNA sequence information into our analysis. Specifically, we retrieved all sequences from the miRBase database [16] and transformed them into vectors using the k-mers method, which divided sequences into subsequences of length $k$, resulting in $m-k+1$ k-mers for a sequence of length $m$. In this study, we extracted adjacent 3-mers from the miRNA sequences. Since miRNAs contain four nucleotides (A, C, G and U), we can split the miRNA sequence into 64 possible combinations, such as AAA, AAC, $\ldots $, UUU. We accomplished this by sliding a window along the miRNA sequence and computing the frequency of each subsequence. Subsequently, we normalized these frequency values to create a 64-dimensional vector that represented the miRNA sequence information, enabling the capture of miRNA attributes.

### Semantic similarity-based attribute feature for diseases

The Medical Subject Heading (MeSH) [17] offers a comprehensive system for disease classification. Building on this, the connections between different diseases are depicted by a directed acyclic graph (DAG). In this graph, nodes symbolize the MeSH descriptors of the diseases, while the directed edges link from broader entities to more detailed ones. For example, a disease $A$ is denoted as $DAG(A) = (D(A), E(A))$, where $D(A)$ represents $A$ and its ancestor nodes, $E(A)$ denotes all the direct edges. Next, we defined the semantic contribution of disease term $t$ in $DAG(A)$: 


(1) \begin{align*}& \left\{\begin{array}{@{}ll} D_{A}(t)=1 & \text{ if}\ t=A \\ D_{A}(t)=\max \left\{\Delta * D_{A}\left(t^{\prime}\right) \mid t^{\prime} \in \text{ children of } t\right\} & \text{ if } t \neq A \end{array}\right.\end{align*}

In this case, $\Delta $ is the semantic contribution’s decay factor, which reduces the impact of disease $t$ when it differs from $A$. Also, disease $A$ keeps a semantic value contribution of 1 by itself.

The semantic value of disease $A$ was as follows: 


(2) \begin{align*}& \mathrm{DV}(\mathrm{A})=\sum_{t \in D(A)} D_{A}(t)\end{align*}

Thus, the disease semantic similarity (DSS1) between diseases $d_{i}$ and $d_{i}$ is first determined through common nodes shared in the two DAGs, calculated as follows: 


(3) \begin{align*}& \operatorname{DSS1}\left(\mathrm{d}_{\mathrm{i}}, \mathrm{d}_{\mathrm{j}}\right)=\frac{\sum_{t \in D\left(d_{i}\right) \cap D\left(d_{j}\right)}\left(D_{d_{i}}(t)+D_{d_{j}}(t)\right)}{D V\left(d_{i}\right)+D V\left(d_{j}\right)}\end{align*}

In addition, we further differentiated the contribution of diseases, as some of them appeared more frequently in other DAGs. Specifically, diseases appeared more often in other DAGs should contribute less compared to those appeared in fewer DAGs. The semantic value of disease $A$ is influenced by disease $t$ in the following way: 


(4) \begin{align*}& D2_{A}(t)=-\log \left(\frac{\text{ the number of DAGs including } t}{\text{ the number of diseases }}\right)\end{align*}

Thus, the disease semantic similarity (DSS2) between diseases $d_{i}$ and $d_{i}$ can also be determined by the common nodes shared in the two DAGs in the following way: 


(5) \begin{align*}& \operatorname{DSS2}\left(\mathrm{d}_{\mathrm{i}}, \mathrm{d}_{\mathrm{j}}\right)=\frac{\sum_{t \in D\left(d_{i}\right) \cap D\left(d_{j}\right)}\left(D2_{d_{i}}(t)+D2_{d_{j}}(t)\right)}{D V\left(d_{i}\right)+D V\left(d_{j}\right)}\end{align*}

Finally, the sum of two semantic similarity model is adopted as the attribute feature of diseases to achieve better prediction performance and solve the cold start problem.

### Gaussian interaction profile kernel similarity-based attribute feature for miRNAs and diseases

We also computed the Gaussian interaction profile (GIP) kernel similarity for miRNAs and diseases, based on the hypothesis that miRNAs with similar functions tend to be related to similar diseases and the other way around. We first represented the association between each miRNA and disease $d(i)$ as a binary vector $G(d(i))$ for diseases. The GIP kernel similarity between diseases $d(i)$ and $d(j)$ ($KD(d(i),d(j))$) was defined as follows: 


(6) \begin{align*}& KD(d(i), d(j))=\exp \left(-\gamma_{d} G(d(i))- G(d(j))^{2}\right)\end{align*}

where parameter $\gamma _{d}$ was the bandwidth of the kernel. It was obtained by normalizing the original parameter $\gamma _{d}^{\prime }$: 


(7) \begin{align*}& \gamma_{d}=\gamma_{d}^{\prime}/\left(\frac{1}{n_{d}} \sum_{i=1}^{n_{d}}\|G(d(i))\|^{2}\right).\end{align*}

We use the same method to calculate the GIP kernel similarity for miRNAs as we do for diseases, which is as follows: 


(8) \begin{align*} & KM(m(i), m(j))=\exp \left(-\gamma_{m} \operatorname{G}(m(i))-G(m(j))^{2}\right) \end{align*}

(9) \begin{align*} & \gamma_{m}=\gamma_{m}^{\prime}/\left(\frac{1}{n_{m}} \sum_{i=1}^{n_{m}}\|G(m(i))\|^{2}\right). \end{align*}

### Integrated attribute features for miRNAs and diseases

To provide a more comprehensive depiction of the attribute features of miRNAs, we concatenated the sequence-based feature ($SM$) and GIP kernel similarity-based feature ($KM$) to designed an integrated miRNA attribute feature matrix $DM$. Specifically, the $DM$ was obtained in the following way: 


(10) \begin{align*}& D M(m(i),m(j)) = SM (m(i),m(j))\oplus KM(m(i),m(j))\end{align*}

Furthermore, an integrated disease attribute feature matrix $DD$ was also designed based on the semantic similarity 1 ($DSS1$), the semantic similarity 2 ($DSS2$) and the GIP kernel similarity of diseases ($KD$). Specifically, the $DD$ was obtained in the following way: 


(11) \begin{align*}& {DD}(d(i), d(j))=\left\{\begin{array}{@{}l} \frac{{DSS1}(d(i), d(j))+{DSS2}(d(i), d(j))}{2}, d(i) \text{ and } d(j) \\ \text{ have semantic similarity } \\{KD}(d(i), d(j)), \text{ otherwise } \end{array}\right.\end{align*}

### Multi-view heterogeneous graphs construction

To excavate the potential associations between miRNAs and diseases that might have been overlooked due to the complexity and heterogeneity of disease pathways, we employed other biomolecules as mediators to construct multi-view graphs. We respectively constructed three heterogeneous graphs, including miRNA–mRNA–disease graph, miRNA–lncRNA–disease graph and miRNA–drug–disease graph. Taking the miRNA–mRNA–disease graph as an example (the remaining two graphs were constructed in the same way), we used different miRNAs, mRNAs and diseases as nodes of the graph, and collected miRNA-mRNA associations and mRNA–disease associations as edges of the graph. Note that MDAs were removed from the graph to prevent label leakage. After that, we represent the miRNA–mRNA–disease graph as an adjacency matrix $MMD$. If there is an association between two items in the matrix, we set the element at the corresponding position to 1; otherwise, we set it to 0. Then, we constructed the miRNA–lncRNA–disease graph and miRNA–drug–disease graph using the same way based on the miRNA-mRNA, mRNA–disease, lncRNA–disease and miRNA-lncRNA associations. We similarly generated the adjacency matrix $MLD$ for the miRNA–lncRNA–disease graph, and the adjacency matrix $MDD$ for the miRNA–drug–disease graph.

### Multi-view graph attention network

To better capture the graph topological features of miRNA nodes and disease nodes in each of the heterogeneous graphs (views) we constructed, we utilized the graph attention neural network for each view. Finally, we fused each miRNA and disease feature in the three views as the final graph structure feature of them with a multi-scale feature fusion module. Taking the miRNA–drug–disease graph as example (the corresponding adjacency matrix $MDD$), we first randomly initialize the feature representation of each miRNA, drug and disease node in the graph as $x=\{x_{1},x_{2},x_{3}, \ldots ,x_{|D|}\},x_{i} \in \mathbb{R}^{F}$, where $|D|$ denoted the node number in the graph and $F$ denoted the dimension of each feature vector. The output of each layer was a new set of graph topology features of the nodes as $x^{\prime }=\{x_{1}^{\prime },x_{2}^{\prime },x_{3}^{\prime }, \ldots ,x^{\prime }_{|D|}\},x_{i}^{\prime } \in \mathbb{R}^{F}$. Generally speaking, the graph attention network first calculated the attention coefficients of adjacent nodes through the self-attention mechanism: 


(12) \begin{align*}& \alpha_{i j}=\frac{\exp \left(\operatorname{LeakyReLU}\left(a^{T}\left[W \cdot x_{i} \| W \cdot x_{j}\right]\right)\right)}{\sum_{k \in N_{i}} \exp \left(\operatorname{LeakyReLU}\left(a^{T}\left[W \cdot x_{i} \| W \cdot x_{k}\right]\right)\right)}\end{align*}

where $W \in \mathbb{R}^{F \times F}$ represents a learnable weight matrix, and $x_{i}$, $x_{j}$ and $x_{k}$, respectively, represents the feature vector of node $i$, $j$ and $k$. The $\cdot $, $\parallel $ and $T$ respectively represents the multiplication, concatenation and transposition operations, $a^{T} \in \mathbb{R}^{2F}$ is the weight parameter of a single-layer feedforward neural network $a$, $LeakyReLU$ is the nonlinear activation function, $exp$ is the exponential function, $N_{i}$ represents all neighbor nodes of node $i$ and $\alpha _{i j}$ represents the attention coefficient from node $i$ to node $j$. To enhance the model’s fitting ability, we then incorporated the multi-head attention mechanism, that is, utilizing multiple $W$ matrices to calculate various attention coefficients simultaneously. The final feature representation of the node was obtained by concatenating the results calculated by each $W$ matrix as follows: 


(13) \begin{align*}& x_{i}^{\prime}=\|_{\phi=1}^{\Phi} \sigma\left(\sum_{j \in N_{i}} \alpha_{i j}^{\phi} W^{\phi} \cdot x_{j}\right)\end{align*}

where $\Phi $ represents the number of $W$ matrix, $\alpha _{i j}^{\phi }$ represents the $\phi $th attention coefficient, $\sigma $ is the activation function and $x_{i}^{\prime }$ represents the final output feature representation of node $i$. Similarly, we, respectively, utilized the graph attention network to extract graph topological features of miRNAs and diseases on the miRNA–mRNA–disease graph and the miRNA–lncRNA–disease graph, which correspond to the adjacency matrices $MMD$ and $MLD$. The difference lied in the dimensionality of the adjacency matrix $MMD$ and $MLD$, i.e., the node number in the miRNA–mRNA–disease and miRNA–lncRNA–disease graph, which are 3929 and 2459, respectively. Furthermore, the learning process of the graph attention network was stopped when the representation of the nodes no longer undergoes large changes, and the final node representations were obtained. Finally, we picked out miRNAs and diseases from each view and concatenated together the output features of the nodes with a dimension of 1778.

### Multi-scale feature fusion module

To effectively fuse three kinds of graph structure features of miRNAs and diseases, so as to more accurately characterize them and improve the prediction accuracy of potential associations, we designed a multi-scale attentional feature fusion module as shown in Figure 1D. Specifically, this module aggregated both the local and global feature context of three kinds of graph structure features of miRNAs and diseases. For aggregating the local channel context of these features, we adopted point-wise convolution (called $PWConv$) method [18], which was a form of convolution that employs a $1\times 1$ kernel and only considered point-wise channel interactions for each spatial position. The local channel context $L(X)$ was calculated by a bottleneck structure as follows: 


(14) \begin{align*}& L(X)=B\left(\mathrm{PWConv}_{2}\left(\delta\left(B\left(\mathrm{PWConv}_{1}(X)\right)\right)\right)\right)\end{align*}

where $X \in \mathbb{R}^{3\times 1778\times 889}$ denoted the original feature matrix of miRNAs and diseases obtained by graph attention neural network (1778 and 889, respectively, denoted the number of miRNAs and diseases and the dimension of the graph structure features). $PWConv_{1}$ and $PWConv_{2}$ respectively denoted two point-wise convolution operations. The kernel sizes of $PWConv_{1}$ and $PWConv_{2}$ were set to $1\times 1778\times 889$ (channel reduction layer) and $3\times 1778\times 889$ (channel increasing layer). $B$ denoted the Batch Normalization (BN). $\delta $ denoted the Rectified Linear Unit (ReLU) function.

For the global channel context $g(X) \in \mathbb{R}^{3}$, the global average pooling (GAP) was utilized as follows: 


(15) \begin{align*}& g(X)=\frac{1}{1778 \times 889} \sum_{i=1}^{1778} \sum_{j=1}^{889} X_{[:, i, j]}\end{align*}

where $i$ and $j$, respectively, denoted the row num and column number of feature matrix $X$. With both the global channel context $g(X)$ and the local channel context $L(X)$, the multi-scale attention mechanism provides the refined feature $X^{\prime } \in \mathbb{R}^{1778\times 889}$ as follows: 


(16) \begin{align*}& X^{\prime}=(X \otimes \sigma(L(X) \oplus g(X)))\end{align*}

where $\oplus $ denoted the broadcasting addition, $\sigma $ denoted the Sigmoid function and $\otimes $ denoted the element-wise dot product operation. The final fusion features of miRNAs and diseases were then fed into an MLP for training and prediction with a standard binary cross entropy loss function.

## RESULTS

### Performance evaluation

**Figure 2:**
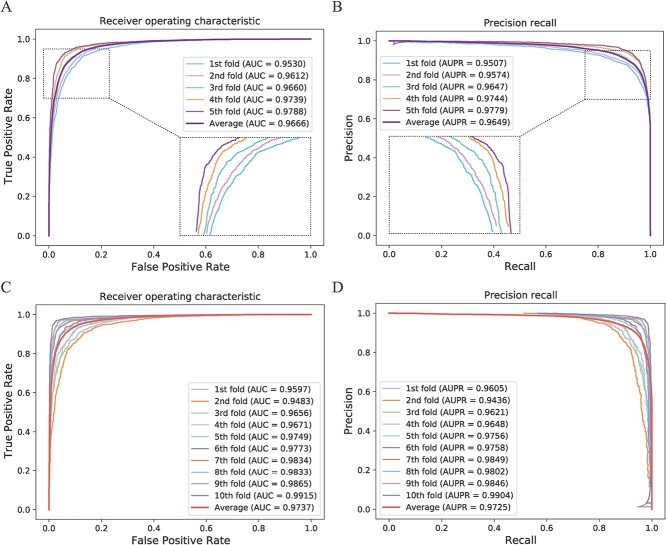
The performance of MUSCLE for 5- and 10-fold CV. (**A**) The ROC analysis results of MUSCLE for 5-fold CV. An enlarged view of the curves is provided in the lower right corner. (**B**) The Precision-recall results of MUSCLE for 5-fold CV. An enlarged view of the curves is provided in the lower left corner. (**C**) The ROCr analysis results of MUSCLE for 10-fold CV. (**D**) The Precision-recall results of MUSCLE for 10-fold CV.

We performed the 5- and 10-fold CV on our model to assess its prediction performance and generalization ability. In the CV, we randomly split all the known MDA into 5 or 10 groups, and then used one of the groups as the test data and the rest as the training data. We repeated this process 5 or 10 times. Finally, we calculated the mean of the test results to evaluate the model. Furthermore, we also plotted the receiver operating characteristic curves (ROCs) and precision-recall curves (PRCs) in the 5- and 10-fold CV to visualize our prediction results (as shown in Figure 2). The area under the ROCs (AUCs) was an evaluation indicator to measure the binary classification model, indicating that the prediction of the probability that positive examples are ranked in front of negative examples. Similarly, the area under the PRCs (AUPRs) was also used as an evaluation indicator. In addition, We also used the other five indicators, including accuracy, sensitive, specificity, precision and Matthews correlation coefficient (MCC), for performance evaluation of our model (as shown in Tables 1 and 2). The average AUC value of MUSCLE under 5- and 10-fold CV can, respectively, reached 0.9666 and 0.9737, and the standard deviation is only 0.0102 and 0.0135. Furthermore, the average AUPR value of MUSCLE also reached 0.9649 and 0.9725, respectively. All these indicators and visualization proved the excellent performance and robustness of MUSCLE for predicting potential MDAs.

**Table 1:** The 5-fold CV performance of MUSCLE

Fold	Accuracy	Sensitivity	Specificity	Precision	MCC	AUC
1st	0.8892	0.8989	0.8796	0.8811	0.7786	0.9530
2nd	0.8965	0.9104	0.8830	0.8829	0.7933	0.9612
3rd	0.9101	0.9083	0.9120	0.9136	0.8202	0.9660
4th	0.9248	0.9227	0.9269	0.9296	0.8495	0.9739
5th	0.9328	0.9338	0.9319	0.9300	0.8656	0.9788
Average	**0.9107 ${\pm }$ 0.0184**	**0.9148 ${\pm }$ 0.0136**	**0.9067 ${\pm }$ 0.0243**	**0.9074 ${\pm }$ 0.0242**	**0.8214 ${\pm }$ 0.0366**	**0.9666 ${\pm }$ 0.0102**

**Table 2:** The 10-fold CV performance of MUSCLE

Fold	Accuracy	Sensitive	Specificity	Precision	MCC	AUC
1st	0.8953	0.9048	0.8858	0.8885	0.7908	0.9597
2nd	0.8858	0.9035	0.8684	0.8705	0.7722	0.9483
3rd	0.9091	0.9137	0.9046	0.9041	0.8183	0.9656
4th	0.9098	0.9218	0.8983	0.8964	0.8199	0.9671
5th	0.9276	0.9268	0.9283	0.9293	0.8551	0.9749
6th	0.9332	0.9275	0.9390	0.9402	0.8664	0.9773
7th	0.9467	0.9388	0.9553	0.9581	0.8934	0.9834
8th	0.9519	0.9592	0.9446	0.9456	0.9039	0.9833
9th	0.9631	0.9652	0.9611	0.9600	0.9262	0.9865
10th	0.9694	0.9699	0.9688	0.9680	0.9387	0.9915
Average	**0.9292 ${\pm }$ 0.0287**	**0.9331 ${\pm }$ 0.0244**	**0.9254 ${\pm }$ 0.0343**	**0.9261 ${\pm }$ 0.034**	**0.8585 ${\pm }$ 0.0573**	**0.9738 ${\pm }$ 0.0135**

### Parameter analysis

To achieve optimal results of classification, we performed a parameter analysis of the MUSCLE method, focusing on two crucial parameters: the embedding dimensions generated by the graph attention network and the number of layers of the MLP. To ensure fairness, we changed only one parameter at a time and kept the other parameters unchanged. Furthermore, to enhance experiment reliability and accuracy for each parameter, the 5-fold CV was conducted. In the following sections, we provided detailed experimental descriptions and results.

#### Impact of embedding dimensions

**Figure 3:**
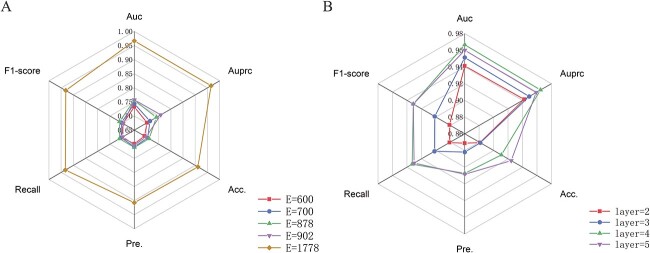
The radar plot for the parameter analysis in MUSCLE. (**A**) The prediction results of MUSCLE on different embedding dimensions of miRNAs and disease nodes generated by the multi-view graph attention network. (**B**) The prediction results of MUSCLE on different hidden layer numbers of the MLP.

We first discussed the impact of the embedding dimensions generated by the multi-view graph attention network. We respectively set the embedding dimensions to (600, 700, 878, 902, 1778), of which 1778 is the sum of the dimensions of attribute features. Table 3 and Figure 3(A) showed the experimental performence for these parameters. It can be seen from the results that as the dimension increases, the predicted AUC of the model continues to increase. When the dimension reaches 1778, the results have been greatly improved. We conjectured that larger feature dimensions provided more information, but the continued increase in feature dimensions would instead increase noise thus causing performance degradation as well as introducing problems such as computational complexity and time overhead.

**Table 3:** Parameter analysis on different embedding dimensions of miRNA and disease nodes

Embedding dimensions	AUC	AUPRC	Accuracy	Precision	Recall	F1-score
E=600	0.7325	0.7022	0.6917	0.6990	0.7020	0.6967
E=700	0.7437	0.7142	0.7051	0.7092	0.7040	0.7029
E=878	0.7564	0.7404	0.7106	0.7114	0.7110	0.7105
E=902	0.7573	0.7584	0.6984	0.7030	0.6971	0.6957
E=1778	**0.9666**	**0.9649**	**0.9107**	**0.9074**	**0.9328**	**0.9313**

#### Impact of MLP Layers

The number of hidden layers of MLP also had a great impact on the prediction result of MUSCLE. We adjusted the hidden layer number from 2 to 5 while maintaining other parameters unchanged. Table 4 and Figure 3(B) showed the experimental results obtained for these parameters. As the number of hidden layers increased, the model’s performance improved. However, as the number continues to increase, the performance decreases due to over-fitting. Based on this, we set the number of MLP to 4.

**Table 4:** Parameter analysis on different hidden layer numbers of the MLP

Hidden layer numbers	AUC	AUPRC	Accuracy	Precision	Recall	F1-score
layer=2	0.9412	0.9423	0.8811	0.8713	0.8810	0.8811
layer=3	0.9513	0.9492	0.8816	0.8819	0.9019	0.9016
layer=4	**0.9666**	**0.9649**	0.9107	0.9074	**0.9328**	**0.9313**
layer=5	0.9600	0.9589	**0.9243**	**0.9085**	0.9302	0.9312

### Ablation experiments

We integrated the biological attribute and three topological features to represent miRNA and disease nodes. We conducted ablation experiments to examine the performance of features in this section. Furthermore, we also examined the validity of our fusion module through the ablation experiments. Similar to the previous experiments, we adopted a control variable method and used the average result for 5-fold CV as the final evaluation metric.

#### Ablation experiment for different features

For the convenience of expression, we defined the attribute features of miRNAs and diseases as $V_{attr}$, and topological features with drugs, mRNAs, and lncRNAs as intermediate nodes as $V_{drug}$, $V_{mRNA}$ and $V_{lncRNA}$. Next, we respectively conducted experiments using different combinations of features and used $+$ to indicate that the corresponding features were considered simultaneously. Table 5 and Table 4(A) showed the performance of MUSCLE with different feature combinations. Finally, the feature combination strategy taken by MUSCLE leads to optimal performance. These heterogeneous topological features enable the model to have a stronger classification performance for potential MDAs.

**Table 5:** Performance comparison of different feature combinations in the ablation experiment

Features	AUC	AUPRC	Accuracy	Precision	Recall	F1-score
$V_{attr}$	0.7907	0.7938	0.7345	0.7414	0.7342	0.7324
$V_{attr + drug}$	0.9397	0.9345	0.8709	0.8713	0.8710	0.8709
$V_{attr + LncRNA}$	0.9401	0.9341	0.8678	0.8683	0.8679	0.8678
$V_{attr + mRNA}$	0.9449	0.9375	0.8746	0.8746	0.8746	0.8746
$V_{drug + mRNA + LncRNA}$	0.9463	0.9450	0.8754	0.8757	0.8754	0.8754
$V_{attr + drug + mRNA + LncRNA}$	**0.9666**	**0.9649**	**0.9107**	**0.9074**	**0.9328**	**0.9313**

#### Ablation experiment for different feature fusion strategies

**Figure 4:**
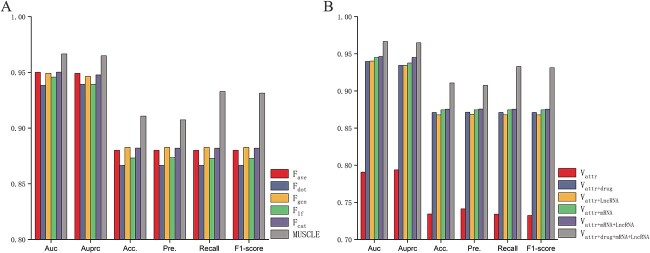
The comparison results of ablation experiments. (**A**) The comparison results between different feature fusion strategies and MUSCLE. (**B**) The comparison results between different topological features and MUSCLE.

To examine the validity of the multi-scale attentional feature fusion module, we conducted ablation experiments using five different feature fusion strategies, including an average value-based feature fusion strategy ($F_{ave}$), a dot product-based feature fusion strategy ($F_{dot}$), a graph convolutional neural network (GCN)-based feature fusion strategy ($F_{gcn}$), a strategy for removing the global features in the multi-scale attentional feature fusion module ($F_{lf}$) and a direct concatenate feature fusion strategy ($F_{cat}$). Table 6 and Figure 4(A) showed the prediction performance of different feature fusion strategies, where our multi-scale attentional feature fusion module (MUSCLE) leads to optimal performance. This appropriate feature fusion strategy enables the model to have a stronger classification performance for potential MDAs.

**Table 6:** Prediction performance of different feature fusion strategies in the ablation experiment

Strategies	AUC	AUPRC	Accuracy	Precision	Recall	F1-score
$F_{ave}$	0.9501	0.9491	0.8801	0.8801	0.8801	0.8801
$F_{dot}$	0.9384	0.9393	0.8666	0.8666	0.8666	0.8666
$F_{gcn}$	0.9491	0.9463	0.8826	0.8826	0.8826	0.8826
$F_{lf}$	0.9457	0.9393	0.8731	0.8735	0.8728	0.8729
$F_{cat}$	0.9502	0.9477	0.8820	0.8819	0.8819	0.8819
MUSCLE	**0.9666**	**0.9649**	**0.9107**	**0.9074**	**0.9328**	**0.9313**

#### Comparison with one single heterogenous graph strategy

**Figure 5:**
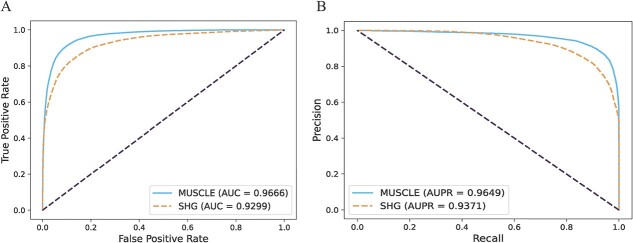
Performance comparison of MUSCLE and one SHG strategy. (**A**) Comparison of ROC curves of MUSCLE and one SHG strategy. (**B**) Comparison of PR curves of MUSCLE and one SHG strategy.

In this work, we respectively constructed three heterogeneous graphs about miRNAs and diseases to extract the relationships between them and other biomolecules from different perspectives. The other option to integrate these relationships is to construct one single heterogeneous graph (SHG) that contains all the different biomolecules. To compare the performance of the two strategies, we reconstructed one SHG with all five different types of biomolecules (miRNAs, drugs, mRNAs, lncRNAs and diseases) and their associations, which integrates three different heterogenous graphs. Note that the SHG generated only one kind of graph structural feature for miRNAs and diseases, so our multi-scale feature fusion module was not available and was discarded in this strategy. Other than that, we kept all the other conditions exactly the same as in our method. Specifically, we first applied the graph attention network to the SHG. We picked out the embedded feature of miRNAs and diseases when the graph attention network converged. To be consistent with our method, we also integrated the same attribute features of miRNAs and diseases into the embeddings from the graph attention network. Finally, the miRNA and disease features were directly fed into the MLP for a 5-fold CV experiment. The training parameters were kept consistent with our method, including the number of iterations: 200, the number of MLP layers: 3, the random number seed: 123, etc. Table 7 shows the comparison results between the SHG strategy and our method with the same evaluation metrics. In addition, we also plot the comparison of ROC curves and PR curves for the two strategies, as shown in Figure 5. From these results, it can be seen that the prediction performance of the strategy with one SHG is not as good as our method. We speculate that the following factors may account for this phenomenon. First, increasing the complexity of the graph may not always result in better feature representation. It is worth exploring pruning or denoising methods on the graph to potentially improve prediction performance. Second, the exclusion of multi-scale feature fusion module may result in suboptimal final feature representations, as evidenced by the ablation experiment on the multi-scale feature fusion module in Ablation experiment for different feature fusion strategies section.

**Table 7:** Performance comparison of MUSCLE with one single heterogenous graph strategy

Fold	Accuracy	Sensitivity	Specificity	Precision	MCC	AUC
1st	0.8542	0.8619	0.8465	0.8489	0.7086	0.9282
2nd	0.8532	0.8616	0.8447	0.8473	0.7064	0.9283
3rd	0.8557	0.8563	0.8551	0.8553	0.7114	0.9309
4th	0.8587	0.8635	0.8540	0.8554	0.7175	0.9310
5th	0.8563	0.8585	0.8542	0.8548	0.7127	0.9311
Average	0.8556 ${\pm }$ 0.0021	0.8604 ${\pm }$ 0.0029	0.8509 ${\pm }$ 0.0049	0.8523 ${\pm }$ 0.0039	0.7113 ${\pm }$ 0.0042	0.9299 ${\pm }$ 0.0015
**MUSCLE**	**0.9107 ${\pm }$ 0.0184**	**0.9148 ${\pm }$ 0.0136**	**0.9067 ${\pm }$ 0.0243**	**0.9074 ${\pm }$ 0.0242**	**0.8214 ${\pm }$ 0.0366**	**0.9666 ${\pm }$ 0.0102**

### Comparison with the state-of-the-art methods

In this section, seven published state-of-the-art (SOTA) methods were selected to compare with our proposed MUSCLE method, including AMHMDA [8], MLRDFM [19], HGSMDA [20], DAEMDA [21], AGAEMD [22], MINIMDA [23] and MAMFGAT [24]. These methods adopt different techniques to predict potential MDAs, including heterogeneous network construction, GCN, feature fusion method, attention mechanism, etc. We briefly introduced the main workflow of these methods in the following list. To be consistent with our method, these baseline methods all meet the following three requirements: (1) Published after 2022. (2) Using the same training dataset as our method, i.e. the Human MicroRNA Disease Database (HMDD v3.2). (3) Using the average result of 5-fold CV method as the evaluation indicator. Table 8 shows the comparison results between our MUSCLE method with these SOTA methods. The experimental evaluation demonstrated that MUSCLE was superior to the recently published methods. It had more promising accuracy and robustness to solve the potential MDA prediction problem. The main workflow of these methods are shown in the follows:

- The AMHMDA method included three main steps for predicting potential associations between miRNAs and diseases. These steps involved constructing multiple similarity networks for miRNAs and diseases, introducing hypernodes to create a heterogeneous hypergraph, and utilizing an attention mechanism to combine the outputs of a graph convolutional network for prediction.
- The MLRDFM method expands upon the DeepFM architecture by improving it in two main ways. Firstly, it includes item relationships by controlling their embedding features through similarity-based Laplacians. Secondly, it utilizes Laplacian eigenmaps to set the weights in the dense embedding layer, leading to more effective model training.
- The HGSMDA method extends upon the HyperGCN model and incorporates the S$\phi $rensen-Dice loss function. It begins by generating networks that capture the similarity between miRNAs and diseases, utilizing GCNs to extract a wide range of information. Subsequently, it forms a miRNA–disease heteromorphic hypergraph with HyperGCN and assesses the accuracy of predicted associations by comparing them to ground truth values using the S$\phi $rensen-Dice loss function.
- The DAEMDA method enhances the efficacy of current models by creating networks that capture the similarity between miRNAs and diseases, including both similarity networks and heterogeneous networks. Leveraging graph attention and self-attention-based feature encoders, it extracts information from neighboring nodes and the entire graph. Finally, it combines node embeddings from dual-channel output and employs an MLP to predict associations between miRNAs and diseases.
- The AGAEMD method utilizes an encoder-decoder framework to predict potential associations between miRNAs and diseases. In the initial phase, it constructs miRNA functional similarity and feature matrices, drawing from disease semantic similarity and the miRNA–disease adjacency matrix. These matrices are subsequently processed by a deep graph attention network, resulting in informative feature embeddings. Finally, an inner product decoder reconstructs the predictive association matrix.
- The MINIMDA method fuses mixed high-order neighborhood information from multimodal networks to predict potential associations between miRNAs and diseases. It constructs integrated miRNA similarity and disease similarity networks using multisource information. The final step involves feeding multimodal embedding representations into a multilayer perceptron to predict underlying associations.
- The MAMFGAT method involves three key steps. Firstly, it constructs MDA and integrated networks. Secondly, the embedded representations of miRNA and diseases are obtained by using a two-path graph attention layer, adaptive modality fusion and modality contrastive learning. Finally, it predicts association scores by connecting these representations through an MLP.

**Table 8:** Performance comparison of MUSCLE with the state-of-the-art methods

Methods	AUC	AUPRC	Accuracy	Precision	Recall	F1-score
AMHMDA [8]	0.9422	0.9411	0.8669	0.8763	0.8549	0.8653
MLRDFM [19]	0.9545	0.9550	0.8833	0.8833	0.8834	0.8833
HGSMDA [20]	0.9481	0.9429	0.8832	0.8806	0.8736	0.8771
DAEMDA [21]	0.9439	0.9429	0.8744	0.8747	0.8763	0.8746
AGAEMD [22]	0.9270	0.9286	0.8502	0.8481	0.8544	0.8507
MINIMDA [23]	0.9446	0.9546	0.8490	0.8547	0.8702	0.8624
MAMFGAT [24]	0.9631	0.9568	0.9001	0.8683	**0.9435**	0.9043
MUSCLE	**0.9666**	**0.9649**	**0.9107**	**0.9074**	0.9328	**0.9313**

**Figure 6:**
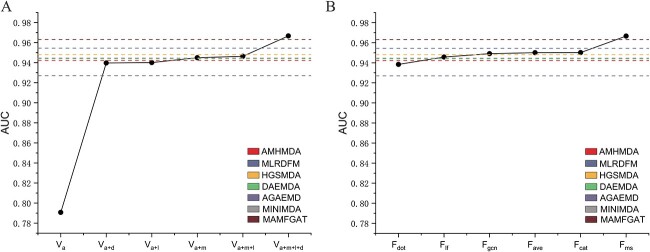
Comparison of the contribution of information increment and algorithmic improvement to our method over the baseline methods. (**A**) The contribution of information increment to our method over the baseline methods. (**B**) The contribution of algorithmic improvement to our method over the baseline methods.

Furthermore, we further clarify whether the performance boosts of our method over some baseline algorithms is due to the information increment or the algorithmic improvement, and what is the contribution of each to the performance boosts. In fact, our data framework and algorithms are an integrated whole, and the algorithms developed based on this data framework. Both the information increment and algorithmic improvement contribute to the performance boosts of our method. First, the increment of information provides more ways to characterize miRNAs and diseases, thereby helping the predictive model make more accurate decisions. Second, the improvement of algorithm makes the characterization more rational, and can benefit the predictive model more from multiple perspectives on the characterization. We have separately compared the contribution of information increment and algorithmic improvement to our method over the baseline methods. The average AUC under 5-fold CV experiment was used as evaluation indicator. First, we control the algorithmic module unchanged and then observe the performance boost of our method over the baseline methods as the information increases (Figure 6A). Second, we control the data module unchanged and then observe the performance boost of our method over the baseline methods as the algorithm improves (Figure 6B). The different colored dotted lines in the Figure represent different baseline methods. The abscissa in Figure 6(A) represents the increase of information, where $V_{a}$ represents only the attribute information of miRNAs and diseases, $V_{a+d}$ represents the addition of drug information on the basis of $V_{a}$, $V_{a+l}$ represents the addition of lncRNA information on the basis of $V_{a}$, $V_{a+m}$ represents the addition of mRNA information on the basis of $V_{a}$, $V_{a+m+l}$ represents the addition of mRNA and lncRNA information on the basis of $V_{a}$, and $V_{a+m+l+d}$ represents the addition of mRNA, lncRNA and drug information on the basis of $V_{a}$. The abscissa in Figure 6(B) represents the improvement of algorithm, where $F_{dot}$ represents the simple dot product of multiple features, $F_{lf}$ represents removing the global attention module in the multi-scale attentional feature fusion module, $F_{gcn}$ represents the use of graph convolutional network instead of graph attention network, $F_{cat}$ represents the simple concatenation of multiple features and $F_{ms}$ represents the use of multi-scale attentional feature fusion. It is clear from the results that both the information increment and algorithmic improvement provide a contribution to the ability of our method to outperform the baseline methods. In addition, the information increment, especially in the initial phase, is more significant in improving our method relative to algorithmic improvement. This reminds us that in future work, we can pay more attention to the improvement of data information and develop new algorithms based on the new data framework. The two parts work together to improve the predictive performance of the model.

### Case study

In order to examine the capacity of MUSCLE in practical applications, we selected three common diseases for case studies, including lung cancer, breast cancer and colon cancer. First, all the known MDAs in our dataset were used for training MUSCLE model. Second, we constructed all except known associations above between miRNAs and corresponding diseases as test dataset. After that, MUSCLE predicted the three test dataset for the corresponding diseases and selected the top 50 miRNAs with the highest predicted scores. Finally, we checked the accuracy of the projected miRNAs using the dbDEMC [25] and miRCancer [26] databases. Case studies of each of the three diseases are detailed below.

Lung cancer is a highly fatal and prevalent malignancy worldwide [27], with non-small cell lung cancer (NSCLC) being the most common type, making up about 85$\%$ of all new lung cancer cases [28]. The researchers had found that miRNAs had a strong association with lung cancer [29]. For example, microRNA-301b enhanced drug resistance, reduced apoptosis and increased cell proliferation in lung cancer [30]. These discoveries not only add to the in-depth study of the development of lung cancer but also present novel targets and therapeutic options for the identification and treatment of lung cancer. Compared with other cancer types, NSCLC is more resistant to chemotherapy, resulting in many patients still facing a poor prognosis after treatment. Therefore, it is very important to predict the possible miRNA biomarkers for the early detection and therapy of lung cancer. The predicted results of MUSCLE are shown in Table 9, where 49 of the top 50 miRNAs were confirmed.

**Table 9:** The top 50 verified miRNAs associated with Lung Cancer

Rank	miRNAs	Evidence	Rank	miRNAs	Evidence
1	hsa-mir-150-5p	dbDEMC	26	hsa-mir-1246	dbDEMC
2	hsa-mir-484	dbDEMC	27	hsa-mir-199a-5p	dbDEMC
3	hsa-mir-486-5p	dbDEMC	28	hsa-mir-377-5p	dbDEMC
4	hsa-mir-629-5p	dbDEMC	29	hsa-mir-449b-5p	dbDEMC
5	hsa-mir-199b-5p	dbDEMC	30	hsa-mir-501-5p	dbDEMC
6	hsa-mir-378a-5p	dbDEMC	31	hsa-mir-34c-5p	dbDEMC
7	hsa-mir-200a-5p	dbDEMC	32	hsa-mir-208b-5p	dbDEMC
8	hsa-mir-106b-5p	dbDEMC	33	hsa-mir-30e-5p	dbDEMC
9	hsa-mir-330-5p	dbDEMC	34	hsa-mir-215-5p	dbDEMC
10	hsa-mir-519b-5p	dbDEMC	35	hsa-mir-23b-5p	dbDEMC
11	hsa-mir-593-5p	dbDEMC	36	hsa-mir-129-2-3p	dbDEMC
12	hsa-mir-621	dbDEMC	37	hsa-mir-636	dbDEMC
13	hsa-mir-28-5p	dbDEMC	38	hsa-mir-885-5p	dbDEMC
14	hsa-mir-106a-5p	dbDEMC	39	hsa-mir-612	dbDEMC;miRCancer
15	hsa-mir-218-2-3p	dbDEMC	40	hsa-mir-135b-5p	dbDEMC
16	hsa-mir-133b	dbDEMC;miRCancer	41	hsa-mir-105-5p	dbDEMC
17	hsa-mir-1469	dbDEMC	42	hsa-mir-3620-5p	dbDEMC
18	hsa-mir-1915-5p	dbDEMC	43	hsa-mir-517c-3p	dbDEMC
19	hsa-mir-193a-5p	dbDEMC	44	hsa-mir-187-5p	dbDEMC
20	hsa-mir-3186-5p	dbDEMC	45	hsa-mir-202-5p	dbDEMC
21	hsa-mir-718	dbDEMC	46	hsa-mir-32-5p	dbDEMC
22	hsa-mir-142-5p	dbDEMC	47	hsa-mir-449a	dbDEMC
23	hsa-mir-154-5p	dbDEMC	48	hsa-mir-764	dbDEMC
24	hsa-mir-194-5p	dbDEMC	49	hsa-mir-675-5p	dbDEMC
25	hsa-mir-632	dbDEMC	50	hsa-mir-1269b	$\mathbf{unconfirmed}$

Breast cancer is a very frequent malignancy among women, and even without taking gender into account, it remains one of the most prevalent cancers after lung cancer. Even though breast cancer has a relatively good prognosis, it still ranks fifth in cancer mortality. High miRNA expression levels have been discovered in breast cancer in recent years, which are linked to worse prognosis for patients. Researchers have found that miRNAs regulate the expression of EZH2 and ATM genes, promote tumor cell proliferation and invasion [31]. The experimental results are shown in Table 10, where 47 of the top 50 miRNAs were confirmed in the database. As an example, hsa-mir-626, a key regulator of tumorigenesis, is expressed at significantly elevated levels in breast cancer. By interacting with miR-573, hsa-mir-626 suppressed the expression of related normal miRNAs and proteins. This implies that hsa-mir-626 could be a possible therapeutic and prognostic target for breast cancer [32].

**Table 10:** The top 50 verified associations associated with Breast Cancer

Rank	miRNAs	Evidence	Rank	miRNAs	Evidence
1	hsa-mir-181a-2-3p	dbDEMC	26	hsa-mir-3611	dbDEMC
2	hsa-mir-486-5p	dbDEMC	27	hsa-mir-4290	dbDEMC
3	hsa-mir-20b-5p	dbDEMC	28	hsa-mir-612	dbDEMC;miRCancer
4	hsa-mir-330-5p	dbDEMC	29	hsa-mir-105-5p	dbDEMC
5	hsa-mir-452-5p	dbDEMC	30	hsa-mir-3620-5p	dbDEMC
6	hsa-mir-593-5p	dbDEMC	31	hsa-mir-340-5p	dbDEMC
7	hsa-mir-503-5p	dbDEMC	32	hsa-mir-576-5p	dbDEMC
8	hsa-mir-718	dbDEMC	33	hsa-mir-626	dbDEMC
9	hsa-mir-154-5p	dbDEMC	34	hsa-mir-519c-5p	dbDEMC
10	hsa-mir-125b-5p	dbDEMC	35	hsa-mir-136-5p	dbDEMC
11	hsa-mir-483-5p	dbDEMC	36	hsa-mir-1275	dbDEMC
12	hsa-mir-204-5p	dbDEMC	37	hsa-mir-212-5p	dbDEMC
13	hsa-mir-632	dbDEMC	38	hsa-mir-517c-3p	dbDEMC
14	hsa-mir-1246	dbDEMC	39	hsa-mir-187-5p	dbDEMC
15	hsa-mir-377-5p	dbDEMC	40	hsa-mir-202-5p	dbDEMC
16	hsa-mir-449b-5p	dbDEMC	41	hsa-mir-449a	dbDEMC
17	hsa-mir-129-2-3p	dbDEMC	42	hsa-mir-764	dbDEMC
18	hsa-mir-583	dbDEMC	43	hsa-mir-542-5p	dbDEMC;miRCancer
19	hsa-mir-1825	dbDEMC	44	hsa-mir-4487	dbDEMC
20	hsa-mir-185-5p	dbDEMC	45	hsa-mir-3151-5p	dbDEMC
21	hsa-mir-491-5p	dbDEMC	46	hsa-mir-511-5p	dbDEMC
22	hsa-mir-636	dbDEMC	47	hsa-mir-1284	dbDEMC
23	hsa-mir-885-5p	dbDEMC	48	hsa-mir-4775	$\mathbf{unconfirmed}$
24	hsa-mir-3651	dbDEMC	49	hsa-mir-147b-5p	$\mathbf{unconfirmed}$
25	hsa-mir-1973	dbDEMC	50	hsa-mir-4447	$\mathbf{unconfirmed}$

Colon cancer is a prevalent gastrointestinal tumor worldwide with a lower 5-years survival rate, and especially in China [33]. Patients detected early have a very high probability of surviving, and with the delay of detection and the aggravation of cancer severity, patients’ survival time would be greatly limited. Therefore, it is very vital to identify colon cancer early and promptly. More and more researches have shown that miRNA is essential for the beginning, progression, and treatment of colon cancer [34]. As shown in Table 11, 48 of the top 50 miRNAs were validated. As an example, Schepeler *et al.* [35] used significance analysis of microarrays (SAM) to detect specific miRNAs, which differentially expressed between colon cancer subtypes and normal mucosa. They discovered that hsa-miR-484 was considerably lower in colon cancer than in normal mucosa.

**Table 11:** The top 50 verified miRNAs associated with Colon Cancer

Rank	miRNAs	Evidence	Rank	miRNAs	Evidence
1	hsa-mir-16-5p	dbDEMC;miRCancer	26	hsa-mir-642a-5p	dbDEMC
2	hsa-mir-29c-5p	dbDEMC	27	hsa-mir-654-5p	dbDEMC
3	hsa-mir-10a-5p	dbDEMC	28	hsa-mir-663a	dbDEMC
4	hsa-let-7g-5p	dbDEMC	29	hsa-mir-769-5p	dbDEMC
5	hsa-let-7i-5p	dbDEMC	30	hsa-mir-302c-5p	dbDEMC
6	hsa-mir-181a-2-3p	dbDEMC	31	hsa-mir-199b-5p	dbDEMC
7	hsa-mir-16-2-3p	dbDEMC	32	hsa-mir-378a-5p	dbDEMC
8	hsa-mir-127-5p	dbDEMC	33	hsa-mir-575	dbDEMC
9	hsa-mir-144-5p	dbDEMC	34	hsa-mir-22-5p	dbDEMC
10	hsa-mir-150-5p	dbDEMC	35	hsa-mir-451a	dbDEMC
11	hsa-mir-30c-5p	dbDEMC	36	hsa-mir-30a-5p	dbDEMC;miRCancer
12	hsa-mir-222-5p	dbDEMC	37	hsa-mir-106b-5p	dbDEMC
13	hsa-mir-20a-5p	dbDEMC	38	hsa-mir-107	dbDEMC;miRCancer
14	hsa-mir-200b-5p	dbDEMC	39	hsa-mir-20b-5p	dbDEMC
15	hsa-mir-34a-5p	dbDEMC	40	hsa-mir-214-5p	dbDEMC
16	hsa-mir-296-5p	dbDEMC	41	hsa-mir-142-5p	dbDEMC
17	hsa-mir-30d-5p	dbDEMC	42	hsa-mir-330-5p	dbDEMC
18	hsa-mir-23b-5p	dbDEMC	43	hsa-mir-452-5p	dbDEMC
19	hsa-mir-365b-5p	dbDEMC	44	hsa-mir-509-3-5p	$\mathbf{unconfirmed}$
20	hsa-mir-484	dbDEMC	45	hsa-mir-4732-5p	dbDEMC
21	hsa-mir-486-5p	dbDEMC	46	hsa-mir-593-5p	dbDEMC
22	hsa-mir-511-5p	dbDEMC	47	hsa-mir-24-1-5p	dbDEMC
23	hsa-mir-518b	dbDEMC;miRCancer	48	hsa-mir-101-2-5p	$\mathbf{unconfirmed}$
24	hsa-mir-615-5p	dbDEMC	49	hsa-mir-29b-1-5p	dbDEMC
25	hsa-mir-629-5p	dbDEMC	50	hsa-mir-424-5p	dbDEMC

## CONCLUSION

In this work, we proposed a computational method (MUSCLE) to predict potential miRNA–diseases associations. First, MUSCLE took a holistic perspective to built a multi-view graph based on the relationships between miRNAs and various biomolecules, including miRNA–drug–disease, miRNA–mRNA–disease and miRNA–lncRNA–disease association graphs. Then, graph attention network was utilized to acquire the graph topology features for each view. Second, MUSCLE efficiently fused multiple graph topology features. Furthermore, MUSCLE also considered the prior attribute knowledge of miRNAs and diseases simultaneously to achieve better prediction results and solve the cold start problem. Finally, the learned representations were then concatenated and fed into an MLP for end-to-end training and predicting. For evaluating the ability of MUSCLE, we respectively conduct 5- and 10-fold CV experiments. MUSCLE outperformed most current state-of-the-art models. Further ablation experiments are performed to verify the efficacy of our feature combination and fusion strategy. Furthermore, the case studies about colon cancer, lung cancer, and breast cancer also fully proved the good inductive capability of MUSCLE. Furthermore, our method still has great potential improvement generalization. For example, our method can be extended to a multi-classification model, which learns and predicts the multiple MDAs at the same time. Besides, the metabolomics information, single-cell sequencing and spatial transcriptome data can be incorporated into the association with miRNA and some specific diseases. These will be the focus of our future work.

**Key Points**

- The MUSCLE method utilizes the relationship among miRNAs, complex diseases and various biological molecules to construct the heterogeneous multi-view graph, and utilizes graph attention network to capture graph topology features.
- The MUSCLE method designs a multi-scale feature fusion strategy to efficiently fuse multiple graph topology features by incorporating the local context into the global context within the attention module.
- The MUSCLE method also considers the prior attribute knowledge of miRNAs and diseases simultaneously to achieve better prediction results and solve the cold start problem.
- The MUSCLE method outperforms most of the existing methods in terms of predictive performance. Each module is optimized to the best, and case studies demonstrate its strong inductive capability.

## Author Biographies

**Boya Ji** is a PhD candidate in the College of Computer Science and Electronic Engineering, Hunan University. His research interests include bioinformatics, deep learning algorithms for biomedical network data.

**Haitao Zou** is a PhD candidate in the College of Computer Science and Electronic Engineering, Hunan University. His research interests include bioinformatics, deep learning algorithms for biomedical network data.

**Liwen Xu** is an associate professor in the College of Computer Science and Electronic Engineering, Hunan University. Her research interests include bioinformatics, deep learning and data mining.

**Xiaolan Xie** is a professor in the College of Information Science and Engineering, Guilin University of Technology. Her research interests include bioinformatics, deep earning and data mining.

**Shaoliang Peng** is a professor in the College of Computer Science and Electronic Engineering, Hunan University. His research interests include biomedical big data, computer aided drug discovery and highperformance computing.
